# Dementia screening: an audit of screening for reversible causes of dementia

**DOI:** 10.1192/bjo.2021.254

**Published:** 2021-06-18

**Authors:** Kim Herbert, Elspeth Richardson, Adam Daly, Christine Carswell

**Affiliations:** 1 NHS Lanarkshire; 2 NHS Greater Glasgow and Clyde

## Abstract

**Aims:**

This audit aimed to assess to what extent patients being referred for assessment of memory problems were receiving appropriate screening for reversible causes. We considered the blood tests recommended by the National Institute for Clinical Excellence (NICE).

**Background:**

Research into ‘reversible dementias’ identified numerous common underlying causes. As a result of this NICE complied comprehensive guidance on investigations which should be performed in the initial stages of assessing patients with memory problems, ideally at a primary care level. These investigations are also crucial at the point of secondary care assessment in order to make a confident diagnosis.

**Method:**

Details of patients referred by their GP to the Older Adult CMHT with memory problems over a one month period were collected. We then used the local laboratory database to note whether each of the eight recommended blood tests had been performed in the preceding 6 months. We measured this against an agreed standard of 95%.

After the first cycle of data collection we prepared business-card sized ‘aide memoirs’ for GPs that could serve as a quick reminder. These were sent out to all GPs in the area along with a letter outlining the audit findings.

**Result:**

Overall 31 patients were included in the first cycle. 15 patients had all 8 dementia blood screens (48%), 13 (42%) had some of the recommended tests and 3 patients had no screening tests at all (10%). On average patients had 76.6% of the recommended bloods completed. The most commonly completed tests were Full Blood Count (FBC) and Urea & Electrolytes (U&Es), with blood Glucose being the most frequently omitted.

In cycle 2, 20 patients were included. Of these patients, 10 had the full complement of screening bloods (50%); 8 had some tests completed (40%) and 2 patients had no screening tests complete (10%). On average 76% of tests were completed. There was an improvement in the rate of completion of both Glucose and Liver Function Tests from cycle1.

**Conclusion:**

This audit demonstrated that current practice does not meet the national standard in general. Our intervention produced a modest improvement in the proportion of patients who received a full complement of dementia screening tests, as well as increasing the rate of patients receiving a blood glucose as part of their screening. It would likely be beneficial to consider further intervention and a 3rd audit cycle in due course.

